# Attitude and opinion towards essential medicine formulary

**DOI:** 10.4103/0253-7613.66837

**Published:** 2010-06

**Authors:** Sangeeta Sharma, Reeta Kh, R. Roy Chaudhury

**Affiliations:** Department of Neuropsychopharmacology, Institute of Human Behaviour and Allied Sciences, Delhi, India; 1 Department of Pharmacology, All India Institute of Medical Sciences, Delhi, India; 2 Department of Patron, Delhi Society for Promotion of Rational Use of Drugs, Delhi, India

**Keywords:** Attitude, clinical practice, essential medicines, formulary, information needs, opinion, rational use of medicines

## Abstract

**Objective:**

The Delhi State Drug Policy was adopted in 1994 following which the first Essential Medicines List (EML) was developed in 1996. The Delhi State Essential Medicines Formulary was brought out in 1997. A need was felt to revise the formulary to match with the EML as the EML is renewed every 2 years.

**Materials and Methods:**

A survey was undertaken to elicit the opinions of the doctors practicing in the state on the usefulness of the formulary before revising and printing the updated version. The survey covered dispensaries, 10–20 bedded hospitals, 100-bedded hospitals and two tertiary care hospitals. Discussions were focused on questionnaires on attitudes toward adopting Essential Medicines Formulary using a 10-point scale.

**Results:**

Of the 200 doctors approached, only 90 doctors completed the questionnaire. Sixty-nine respondents (76.6%) had received the copy of the formulary. Most practitioners welcomed the formulary and were satisfied with the coverage and selection of the medicines. Most respondents (76.9%) agreed that a well-developed formulary would improve the quality of the public health care system, although they had reservations about the authority, relevance and effect on professional autonomy.

**Conclusion:**

About 74% of the respondents used the formulary in clinical practice as a source of medicine information, which makes its regular revision necessary.

## Introduction

Access to clinically relevant, up-to-date, user-specific, objective and unbiased medicine information is essential for appropriate medicine use and is a basic requirement for rational medicine practice. The Delhi State Drug Policy was adopted in 1994 and the first Essential Medicines List (EML) was developed in 1996, which is revised every 2 years. The EML and a centralized pooled procurement system resulted in better accessibility to medicines.[1] More than 90% of the patients got quality medicines prescribed to them in public hospitals.[2–4] However, the prescribing practices were not consistent with the rational use of medicines and were contributing to wastage of meager resources.[1–3] Because medicine information is vital for any country and health institutions striving to rationalize treatment, the first Delhi State Essential Medicines Formulary (EMF) was developed and distributed to medical doctors of public health facilities in 1997. A great deal of unpaid time and effort goes into the production of such formularies, yet their effectiveness and appropriateness has not been adequately established.[4] As with treatment guidelines, the production of an EMF is an ongoing process. Hence, before undertaking further revision and printing, this study was planned to elicit the attitude and opinion of the doctors about its usefulness, and suggestions were sought as formularies ought to reflect the wishes and needs of the users.

## Materials and Methods

In the prospective survey, doctors working at various levels of healthcare, i.e., dispensaries, 10–20-bedded hospitals, 100-bedded hospitals and two tertiary care hospitals were approached. Interview guides were designed to progress from open-ended inquiries to specific responses. Discussions were focused by 12 questions, including seven items adapted from the Mansfield’s (1995) questionnaire[5] on attitudes toward the Formulary[3] using a 10-point strongly agree–strongly disagree scale. This also included questions on information needs of the practitioners using a five-point first priority–last priority scale and suggestions for improvement in the formulary.

## Results

A total of 200 doctors were approached, of which 90 completed the questionnaire. The others were reluctant to contribute due to time constraints and work pressure. Sixty-nine respondents (76.6%) had received the copy of EMF and had gone through it. Of the 69 respondents, 18.8% represented tertiary care hospitals, 46.4% secondary care hospitals and 34.8% dispensaries. The respondents from tertiary care were consultants and senior residents from various specialties. Of the 69 respondents, 65.2% stated that all the essential medicines (EM) were included and 57.97% stated that the coverage/details of these EM was satisfactory, and 10.14% felt that not all the EM were included, like medicines for critical care, antiretroviral agents and medicines to be given during pregnancy.

Practitioners referred to the EMF occasionally (60.86%), weekly/fortnightly (8.69%) and on a daily basis (4.34%), whereas 2.89%, although in possession of the EMF, never used it. The median attitude scores are shown in Table 1. Most practitioners (94.5%) had a welcoming attitude toward the EMF and found it to be moderately to extremely useful. A few (14.5%) were unable to comment on their colleagues’ attitudes (how am I supposed to know). Of the doctors who did give a score for their colleagues, 34.5% scored their colleagues as equally extremely welcoming; however, 7.3% of the practitioners stated that the attitude of their colleagues toward the EMF was resentful. About 56.5% stated that they are not reluctant to use these EM in their daily practice. Although most practitioners (76.9%) strongly agreed that a well-developed EMF would improve the quality of public health, 24% of the practitioners had reservations as they felt that using EMF medicines reduces their autonomy and 16.3% felt that it restricts flexibility and thus denies individuality of the patient care.

**Table 1:** Attitude toward formulary in clinical practice (median score is underlined)

1. How useful do you believe the formulary is to you in your clinical practice?	Extremely useful 1	2	3	4	5	6	7	8	9	Totally useless 10
2. How would you describe your attitude towards the formulary	Extremely welcoming 1	2	3	4	5	6	7	8	9	Extremely resentful 10
3. How would you describe the attitude of most of your colleagues towards formulary	Extremely welcoming 1	2	3	4	5	6	7	8	9	Extremely resentful 10
4. Would you be reluctant to use essential medicines that were aided at reducing costs without affecting patient outcomes?	Extremely reluctant 1	2	3	4	5	6	7	8	9	Not at all reluctant 10
5. Do you agree or disagree that using essential medicines reduces the autonomy of doctors?	Strongly agree 1	2	3	4	5	6	7	8	9	Strongly disagree 10
6. Do you agree or disagree that essential medicines deny the individuality of the patient?	Strongly agree 1	2	3	4	5	6	7	8	9	Strongly disagree 10
7. Do you agree or disagree that the implementation of well developed formulary would improve the quality of care within the public health system?	Strongly agree 1	2	3	4	5	6	7	8	9	Strongly disagree 10

In terms of qualitative responses, most respondents considered EMF as a good aid for quick access to details such as indications of therapeutic use and dosage range about commonly prescribed medicines. The information provided in the EMF is precise, unbiased and simple, making it reader-friendly and handy. Most respondents (68%) did not offer any suggestions for improving the EMF. A few respondents felt that information should be provided about brand names, price of the medicines, practical prescribing in critical care, surgical prophylaxis and infections, dermatological infections and management for adverse-effects, interactions and choice of medicines in pregnancy and in liver and kidney disease along with doses [Table 2]. Median scores of the information needs of the practitioners are shown in Table 3. Most respondents preferred, in order of priority, to receive information on recent advances and new medicines released in the market, adverse drug reactions, drug interactions and microbiological resistance pattern.

**Table 2:** Suggestions of the doctors for improving the next edition of essential medicine formulary

*Value label*	*Frequency (n = 69)*	*Percentage*
No comments	47	68.11
Include medicine pricing information	3	4.34
Include brand names	3	4.34
Keep it updated	5	7.24
Include more information on practical prescribing in critical care, surgical prophylaxis and infections, dermatological infections and treatment for adverse-effects	5	7.24
Include medicines interactions	1	1.44
Include dosage schedules in pregnancy and diseases	4	5.79
Include more number of medicines from antiretroviral, anticancer medicines	2	2.89
Include doctors from various specialties in the panel	1	1.44

**Table 3:** Median scores of the information needs of the practitioners (median score is underlined)

*Information need*	*First priority*				*Last priority*
Adverse drug reaction reports	1	2	3	4	5
Drug interactions	1	2	3	4	5
New medicines released	1	2	3	4	5
Microbiological resistance	1	2	3	4	5
Recent advances in therapeutics	1	2	3	4	5

## Discussion

A questionnaire was administered to doctors to learn about their experience with and attitude toward the EMF, and the response rate was 45%. Although not a representative sample, the response rate reflects that doctors have an overall welcoming attitude toward the EMF, and about 74% referred to it in their practice occasionally to daily. Previous similar surveys have received an approximately 38–70% response rate,[4,6,7] which were lower than that received in the present study. The non-responders constitute a major problem in such surveys as one cannot assume that they are not interested in formularies or do not use formulary.

Most respondents, particularly in primary care, considered EMF as a good aid for quick access to details such as indications of therapeutic use and dosage range about commonly prescribed medicines. Other studies have also reported as positive opinion about medicine formulary by general practitioners in the primary care and have shown that the use of the formulary changed the prescribing habits.[8,9]

In the present study, although most practitioners strongly agreed that a well-developed formulary would improve the quality of the public health care system, some from the tertiary care had reservations that using formulary medicines reduces their autonomy and restricts flexibility and individuality of the patient. Similar results have been reported in a study that showed that the attitude toward formularies in general was negative. The practitioners agreed that formularies increased the amount of time spent in making medicine choices, resulted in less-effective medicines, compromised the quality of medicines prescribed and reduced the opportunities to offer the best medication for patients.[9,10]

The general presentation and selection of EM in EMF was acceptable to most respondents. The results suggest that there is little demand to change the presentation of the EMF. Respondents in the present study wanted information on the prices of medicines in public vis-a-vis private sector. Higher medicine prices have been shown to negatively impact patient outcomes. Studies suggest that doctors have a poor understanding of the pharmaceutical pricing mechanics, but the data are variable and there is no consistent pattern in awareness.[11,12] Highly priced medicines were estimated more accurately than inexpensive ones (74% versus 31%). Doctors consistently overestimated the price of inexpensive products and underestimated the expensive ones. When asked, they expressed that they wish to have price information and felt that this would improve their prescribing but not accessibility.[12] However, providing price data of all the brands in a formulary as expected by the respondents may not be feasible, given the frequent changes in medicine pricing, number of brands and wide variation in prices of different brands available in the market, and whether it is worth the effort for a formulary with an expected life of 2 years. However, prices paid in the public health facilities compared to price range with minimum, maximum and median in the market could be provided in the EMF.

Regarding information needs of the practitioners, medicine interactions and adverse drug reactions are addressed in the formulary, but there is a limitation to provide information about new medicines released in the market and recent advances because the medicines formulary contains information only on medicines that are in the market for a considerable amount of time (usually 3–5 years) for which sound and adequate data on efficacy and safety are available.[13] New medicines are selected by the committee for selection of EM balancing criteria of efficacy, safety, suitability, quality and cost. The present study revealed that almost 74% of the respondents used the EMF as a source of medicines information, and this makes its regular revision important.
